# Morally Injurious Experiences and Emotions of Health Care Professionals During the COVID-19 Pandemic Before Vaccine Availability

**DOI:** 10.1001/jamanetworkopen.2021.36150

**Published:** 2021-11-24

**Authors:** Ye Kyung Song, Sneha Mantri, Jennifer M. Lawson, Elizabeth J. Berger, Harold G. Koenig

**Affiliations:** 1 Department of Psychiatry and Behavioral Sciences, Duke University Medical Center, Durham, North Carolina; 2 Department of Neurology, Duke University Medical Center, Durham, North Carolina; 3 Trent Center for Bioethics, Humanities, and History of Medicine, Duke University, Durham, North Carolina; 4 Department of Pediatrics, Duke University Medical Center, Durham, North Carolina; 5 Donald and Barbara Zucker School of Medicine at Hofstra/Northwell, Hempstead, New York; 6 Department of Medicine, King Abdulaziz University, Jeddah, Saudi Arabia

## Abstract

**Importance:**

Moral injury in health care professionals (HPs) has worsened over the course of the COVID-19 pandemic. The trauma and burnout associated with moral injury has profound implications for the mental health of HPs.

**Objective:**

To explore the potential factors associated with moral injury for HPs who were involved in patient care during the COVID-19 pandemic in 2020, prior to the availability of vaccines.

**Design, Setting, and Participants:**

In this qualitative study, HPs were actively recruited to participate in a survey via snowball sampling via email and social media in 2 phases of 5 weeks each: April 24 to May 30, 2020 (phase 1), and October 24 to November 30, 2020 (phase 2). Overall, 1831 respondents answered demographic questions and assessments for moral injury, intrinsic religiosity, and burnout. Of those, 1344 responded to the open-ended questions. Responses to open-ended questions were coded iteratively and thematically analyzed within the framework of moral injury.

**Exposures:**

Working in a patient care setting during the COVID-19 pandemic prior to the availability of vaccines.

**Main Outcomes and Measures:**

Inductive thematic analysis of open-response survey answers identified dominant emotions and common stressors associated with moral injury.

**Results:**

There were 335 individuals (109 [32.6%] aged 35-44 years; 288 [86.0%] women; 294 [87.8%] White) in phase 1 and 1009 individuals (384 [38.1%] aged 35-44 years; 913 [90.5%] women; 945 [93.7%] White) in phase 2. In phase 1, the respondents were predominantly nurses (100 [29.9%]), physicians (78 [23.3%]), advanced practice practitioners (APPs) (70 [20.9%]), and chaplains (55 [16.4%]). In phase 2, the respondents were predominantly nurses (589 [58.4%]), physicians (114 [11.3%]), and APPs (104 [10.3%]). HPs faced numerous stressors, such as fear of contagion, stigmatization, short-staffing, and inadequate personal protective equipment. The emotions experienced were (1) fear in phase 1, then fatigue in phase 2; (2) isolation and alienation; and (3) betrayal.

**Conclusions and Relevance:**

These findings suggest that HPs experienced moral injury during the COVID-19 pandemic. Moral injury was not only experienced after a single moral dilemma but also from working in morally injurious environments. These experiences can serve as potential starting points for organizations to engender and enhance organizational and individual recovery, team building, and trust. System-level solutions that address shortages in staffing and personal protective equipment are needed to promote HP well-being.

## Key Points

**Question:**

How did health care workers experience moral injury during the COVID-19 pandemic before the availability of vaccines?

**Findings:**

In this qualitative study of 1344 health care professionals in 2020, respondents reported significant changes in their personal and professional lives during the course of the pandemic. Common themes were feeling isolated from non–health care professionals, alienated from patients, and betrayed by coworkers, administrators, and the public.

**Meaning:**

Moral injury can result from chronic stressors in morally injurious environments; leadership must identify and address these stressors to effectively support health care professionals as COVID-19 continues to strain staff’s physical, mental, and emotional resources.

## Teaser

This qualitative study explores the factors associated with moral injury for health care workers who were involved in patient care during the COVID-19 pandemic in 2020, prior to the availability of vaccines.

## Introduction

Moral injury is emotional distress resulting from events or transgressive acts that create dissonance within one’s very being due to a disruption or violation of their existential orientation and values system.^1^ There may even be betrayal against what is held to be right by someone who holds legitimate authority in a high-stakes situation.^2^ When synthesized, a holistic conceptualization of moral injury examines both the personal actions and beliefs of individual actors but also takes into account a society’s actions and beliefs in the world from a historical and sociological point of view.^3^

Initially conceived in the context of combat trauma and the experiences of war veterans,^4^ moral injury has been theorized and studied in the context of health care professionals (HPs).^5,6,7^ Moral injury manifests itself as a double bind: “[d]o we take care of our patient, the hospital, the insurer, the EMR [electronic medical record], the health care system, or our productivity metrics first? There should only be 1 answer to that question, but the current business framework of medicine pressures us to serve all these masters at once.”^8^

Moral injury is strongly associated with medical errors, clinician burnout, and increased suicidal thoughts.^9,10^ Prior to the COVID-19 pandemic, nearly 1 in 4 HPs reported at least moderate impairment in family, social, or occupational functioning.^9^ Both mental health disorders and burnout are projected to increase among clinicians in the wake of the pandemic^11,12,13^; early evidence suggests that increases in moral injury may be a bellwether of a workforce under tremendous strain.^14^

The challenges reported in past pandemics included increased workload amid new and frequently changing protocols and fractured interpersonal relations complicated by personal protective equipment (PPE).^15,16,17,18,19,20,21,22^ These systemic stressors have a direct association with HP moral injury, distress, and burnout.^14,23^ However, there are few empirical studies that qualitatively describe the emotional experiences and perspectives of HPs. The purpose of this study is to understand the experiences of HPs during the COVID-19 pandemic within the previously mentioned framework of moral injury.

## Methods

This qualitative study used a grounded theory approach^24^ and follows the Consolidated Criteria for Reporting Qualitative Research (COREQ) reporting guideline.^25^ This study was reviewed by Duke Health’s institutional review board and deemed exempt. The methodology used to collect the qualitative data analyzed in this cross-sectional qualitative study is described in detail by Mantri et al.^14^ In summary, HPs were recruited via snowball sampling through email distributions and social media platforms. Recruitment was conducted in 2 phases of 5 weeks each: April 24 to May 30, 2020 (phase 1), and October 24 to November 30, 2020 (phase 2). Participants who clicked the link were taken to the front page, which was a written informed consent statement. Participants clicked “I consent, begin the study” to continue. Overall, 1831 respondents completed the survey, and of those, 1344 respondents answered the following open-ended questions:

1. Think of a time during this pandemic when you were caring for someone with COVID-19. This can be a family member, a patient, or a friend. What was that experience like for you?
2. How has the pandemic affected your interactions with people at work? At home? At any community spaces you are part of?
3. What has been your greatest source of fear during the pandemic?
4. What has sustained you?
5. Is there anything else you would like to tell us about your personal or professional experiences with COVID-19?
6. [Phase 2] How has your response to COVID changed since March 2020?

There was no limit to the amount of time that participants could spend on the survey, and there was also no word or character limit. To protect confidentiality, participants did not provide any personally identifying information and responses were assigned a randomly generated identifier.

### Data Analysis

Only those who completed at least 1 open-ended question were included in the qualitative analysis. Demographic characteristics of these participants appear in Table 1. NVivo version 12 (QSR International) was used to store the data obtained from the survey and to create and organize codes. We coded all free-text responses while blinded to the participant’s demographic characteristics, Moral Injury Symptoms Scale–Healthcare Professionals (MISS-HP; 10-item scale assessing symptoms of moral injury among HPs; range, 10-100, with higher scores indicating greater moral injury), Duke University Religion Index (DUREL; a 5-item measure of religiosity; range, 3-15, with higher scores indicating greater religiosity), and abbreviated Maslach Burnout Inventory (aMBI; a 9-item scale assessing emotional exhaustion, depersonalization, and reduced personal/professional accomplishment; subscale range, 0-18, with higher scores indicating greater burnout on the exhaustion and depersonalization subscales and higher scores indicating less burnout on the accomplishment subscale) scores to mitigate coding bias. The codes were generated in an iterative process. After contextualizing coded responses by unblinding demographic characteristics and MISS-HP, DUREL, and aMBI scores, these codes were then grouped into larger thematic categories in an inductive method (Table 2).

**Table 1.** Demographic Characteristics of Participants Who Completed the Qualitative Questions

Characteristic	Respondents, No. (%)^a^	
	Phase 1 (n = 335)	Phase 2 (n = 1009)
Age, y		
18-24	8 (2.4)	16 (1.6)
25-34	89 (26.6)	225 (22.3)
35-44	109 (32.6)	384 (38.1)
45-54	69 (20.6)	245 (24.3)
55-64	41 (12.2)	121 (12.0)
65-74	19 (5.7)	15 (1.5)
≥75	0	3 (0.3)
Gender		
Male	43 (12.8)	94 (9.4)
Female	288 (86.0)	913 (90.5)
Nonbinary	4 (1.2)	2 (0.2)
Race		
White	294 (87.8)	945 (93.7)
Black	13 (3.9)	8 (0.8)
American Indian or Alaska Native	5 (1.5)	11 (1.1)
Asian	19 (5.7)	31 (3.1)
Hawaiian or Pacific Islander	0	6 (0.6)
Other^b^	14 (4.2)	36 (3.6)
Marital status		
Married	229 (68.4)	665 (65.9)
Widowed	1 (0.3)	18 (1.8)
Divorced	33 (9.9)	130 (12.9)
Separated	4 (1.2)	14 (1.4)
Never married	68 (20.3)	182 (18.0)
Religion		
Christian	232 (69.3)	688 (68.2)
Jewish	15 (4.5)	16 (1.6)
Hindu	6 (1.8)	5 (0.5)
Muslim	3 (0.9)	3 (0.3)
Buddhist	6 (1.8)	4 (0.4)
Atheist/agnostic	46 (13.7)	205 (20.3)
Other	27 (8.1)	88 (8.7)
Profession		
Nurse	100 (29.9)	589 (58.4)
Physician	78 (23.3)	114 (11.3)
APP	70 (20.9)	104 (10.3)
Housekeeping	0	0
Food services	0	0
Interpreter	0	1 (0.1)
Patient transport	0	0
Chaplaincy	55 (16.4)	1 (0.1)
Social worker	11 (3.3)	17 (1.7)
Other	21 (6.3)	183 (18.1)
Geography		
United States		
Northeast	56 (16.7)	130 (12.9)
Mid-Atlantic	25 (7.5)	44 (4.4)
Southeast	153 (46.7)	193 (19.1)
Midwest	51 (15.2)	376 (37.3)
Southwest	21 (6.3)	122 (12.1)
Pacific Northwest	19 (5.7)	108 (10.7)
Canada	4 (1.2)	26 (2.6)
Central America	0	2 (0.2)
South America	0	0
Western Europe	2 (0.6)	5 (0.5)
Eastern Europe	1 (0.3)	1 (0.1)
Africa	0	1 (0.1)
China	0	0
Other Asia	1 (0.3)	2 (0.2)
Oceania	2 (0.6)	14 (1.4)
Coronavirus experience statements		
I currently care for COVID-19 patients on a regular basis	114 (34.0)	475 (47.1)
I currently care for COVID-19 patients intermittently (eg, backup care)	77 (23.0)	239 (23.7)
I have cared for COVID-19 patients in the past but not currently	20 (6.0)	125 (12.4)
I have personally become ill from SARS-CoV-2/COVID-19	10 (3.0)	94 (9.3)
I have a family member who has become ill from SARS-CoV2/COVID-19	32 (9.6)	209 (20.7)
I have not had personal or professional experience caring for patients with SARS-CoV-2/COVID-19	72 (21.5)	75 (7.4)
I currently care for non–SARS-CoV2/COVID-19 patients	134 (40.0)	318 (31.5)
I am not currently involved in patient care	25 (7.5)	52 (5.2)

Abbreviation: APP, advanced practice practitioner.

^a^ Percentages for race and geography categories may add to greater than 100%, as respondents could select multiple options.

^b^ Participants self-selected the “other” category.

**Table 2.** Coding Schema

Theme	Codes
Fear	Anxious, worried, concerned
Fatigue	Demoralizing, challenging, exhausted, drained
Isolation and alienation	Bizarre, chaotic, interesting, guilty, heartbroken, helpless, hopeless, isolated, overwhelmed, sad, difficulty communicating/connecting due to PPE
Betrayal and community	Angry, abandoned, frustrated, grateful, proud, honored, finding meaning, trapped, traumatic, under-prepared

Abbreviation: PPE, personal protective equipment.

Themes were first identified in phase 1 and phase 2 separately, then compared across phases to examine change over time. Phase 1 captured the emergence of the pandemic in the United States, and phase 2 captured the continuation of the pandemic, before vaccine distribution.

## Results

A total of 1344 HPs completed the qualitative portion of the survey: 335 individuals (109 [32.6%] aged 35-44 years; 288 [86.0%] women; 294 [87.8%] White) in phase 1 and 1009 individuals (384 [38.1%] aged 35-44 years; 913 [90.5%] women; 945 [93.7%] White) in phase 2. In phase 1, the respondents were predominantly nurses (100 [29.9%]), physicians (78 [23.3%]), advanced practice practitioners (APPs) (70 [20.9%]), and chaplains (55 [16.4%]). In phase 2, the respondents were predominantly nurses (589 [58.4%]), physicians (114 [11.3%]), and APPs (104 [10.3%]). Additional demographic information is presented in Table 1.

The responses across both phases were grounded in and expressed through emotions related to changes in family, social, and occupational functioning. The primary themes were (1) fear in phase 1, then fatigue in phase 2; (2) isolation and alienation; and (3) betrayal/community. Exemplar responses are presented in eAppendices 1 to 3 in the Supplement.

### Fear to Fatigue

The predominant emotions shifted between the 2 phases. In phase 1, most participants expressed fear and uncertainty about the virus itself and its societal consequences. Fears were predominantly associated with “catching the virus” and becoming ill and/or “spreading it” to friends, family, and patients. Some referenced fear of COVID-19 transmission to higher risk people, such as pregnant people, older people, and/or those with other medical comorbidities. Part of this fear was due to the idea that those with COVID-19 dying “isolated and somewhat neglected … I know that COVID patients are not getting the care we’d give, say, a person with the flu, for the safety of the HPs” (respondent 2). Witnessing patients die alone due to isolation measures was “heartbreaking” and also “has also shaken my faith in medicine, it makes me feel vulnerable and scared … there isn’t much modern medicine can do to help” (respondent 2).

In phase 2, most participants stated that as there was more knowledge about COVID-19, there was a decrease in fear: they were “over it” and experiencing “COVID fatigue.” There was also resignation around adapting to “the new normal.” One nurse described how “it’s routine work. You get used to people dying and gowning/masking. It’s almost like we have been conditioned at this point” (respondent 1).

In phase 1, the reliability and scarcity of PPE was also a source of fear, as some doubted its safety and its continued availability. The constantly changing protocols from one day to the next raised doubts that HPs were adequately protected: “We just all assume that we will get sick or have asymptomatic COVID at some point” (respondent 3). To some degree, the questioned safety of PPE led to an attempt to reduce exposures, and primary ownership of the patient fell onto nursing staff “while everyone else watches from afar and refuses to help to ‘lessen exposure’” (respondent 3). In phase 2, many respondents stated that as their PPE became more available with better protocols in place, their fear is gone and replaced with exhaustion: “my levels of fear have decreased, just because they weren’t sustainable” (respondent 4). More knowledge was often associated with less fear, becoming more lax with PPE, and slowly resuming normal activities.

### Isolation and Alienation

Across phase 1 and phase 2, many reported feeling isolated and lonely. Respondents described experiences of becoming distrustful and afraid of others, including patients and coworkers “who have lied and hid in [*sic*] symptoms ultimately exposing me, my patients, and clinic staff” (respondent 5). “I don’t know that others are taking it as seriously as I am” (respondent 6). As previously stated, many were afraid of putting others at risk; as such, they circumvented social situations to avoid “feeling like the dirtiest girl at the party” (respondent 6). Some noted that others have become afraid of HPs, including family, friends, and people in the community, leading to some to avoid running errands “even before work if I’m in scrubs because of [the stigma]” (respondent 7).

Some people isolated and withdrew physically from others “for fear of infecting them or exposing them to this as every day I feel that I am a carrier” (respondent 8). They also withdrew emotionally, often assuming other people did not know what they were going through. People felt isolated from their community due to politicized discourses circulating on social media on social distancing protocols, such as wearing masks, especially after having “dealt with death or even just the difficulty of caring for these patients” (respondent 9). One respondent claimed it as their “most painful moral injury … fighting hard at front lines and coming home from gnarly ER shifts only to have to battle on social media” (respondent 10).

While physically present at work, masks and social distancing contribute to feelings of isolation. Many stated that it was stressful adapting to social distancing measures, such as having to “stand farther away from a colleague than previously” (respondent 11), not being able to see the entirety of facial expressions, or even recognizing coworkers in the hallway. The close interactions that would have bonded coworkers together are restricted, and many outlets for stress have been suspended.

Many found video conferencing interactions to be “just not the same,” and “work hours and time to decompress/rest after shifts makes even a virtual social life almost impossible” (respondent 12). One respondent stated, “Life is a Zoom meeting, and completely unrewarding” (respondent 13). Despite the sentiment that virtual interactions are not the same as in-person ones, some did find the incorporation of technology to be positive, engaging more with family, coworkers, and the community.

Across both phases, the use of PPE and social distancing measures in patient care contributed to isolation from patients, with HPs feeling as if they were providing suboptimal care. As essentialized by one respondent, “We can’t build a connection with our patients because we can’t spend the time to really care for them the way they deserve to be cared for” at the bedside (respondent 14). Many lamented the use of telehealth in clinics and loss of “the true connection,” finding telehealth to be “dehumanizing and disjointed” (respondent 15). Being unable to enter patient rooms has also been a struggle, as many stated feeling “less than helpful and effective patient care. We are a disjoined voice at the end of the phone line … how could we possibly help?” (respondent 16). As alluded to previously, some respondents felt guilty about not being able to be physically present for the patient, as well-being positioned them at a distance from the frontlines.

Being on the frontlines and physically present with the patient meant donning and doffing PPE for protection. The donning and doffing process contributed to the stress and panic on the hospital unit: “We’re trying to help patients in one room but call bells are going off in another room and you can’t get there. More than a few times patients have crawled out of bed and fallen because you can’t get there fast enough” (respondent 17). In some ways, it was alienating and surreal, “like being on a game show. You’re put into crazy PPE that you have to keep on for prolonged periods of time, sometimes have to work in unknown environments with unfamiliar coworkers with extremely sick patients with doctors who might run out of options in the care for the extremely sick. It was a harrowing experience and you had to adapt to this new environment or not swim” (respondent 18).

Many affirmed this sentiment that working during COVID was more about survival: “We all have built walls to protect ourselves and survive ICU [intensive care unit]. COVID made those walls thicker, stronger, impenetrable. We get lost, unable to reach out, to feel things. We have turned to stone” (respondent 19). Being the sole physically present support for patients who are ill and dying has been described as heartbreaking: “I didn’t let them die alone … I am an angel of death and comfort. That weighs on me” (respondent 20).

### Betrayal/Community

In phase 1, early in the pandemic, there was an inadequate supply and/or questionable PPE. Many institutions recycled N95 masks and provided PPE that was labeled not for medical use. Some employees were welcomed to bring their own to work in as “good stewards of PPE.” One respondent stated, “I felt like our lives were more disposable than our PPE was” (respondent 21). Another stated, “I felt as though we were being ‘offered up for slaughter’ by having to stay in a COVID filled room with questionable PPE” (respondent 22). This sentiment did not diminish even in phase 2, several months into the pandemic when these entities ostensibly had time to shore up resources and provide adequate protection and support. HPs felt abandoned: “We’ve just gotten better at protecting ourselves. Our hospital doesn’t do a lot for us” (respondent 23).

HPs felt betrayed and unsupported by management, administrators, institutions, the health care system more broadly, and the government. Many pointed to a disconnect between leadership and “those of us in there doing the hard work.” “During it all, our leadership had a tendency to focus on all we were doing wrong if they were there when they talked to us, instead of what we were doing right. I realized early on that we had to look after our team ourselves. I think the relationship between the doctors and nurses became that much better. We all had a greater appreciation of what each other did as we spent so much time together” (respondent 24).

As reflected in the previous quote, many were able to find a sense of community with their coworkers through the experience of shared trauma. The common goal of providing patient care also bonded coworkers together while creating and deepening divides between others. “There were coworkers who I really trust, and other coworkers who I am deeply distrustful of … I no longer trust the management of my unit or hospital” (respondent 25).

## Discussion

This study contributes to the research on HPs in COVID-19, as it is one of the few that is grounded in the emotional salience of being at the center of a global pandemic. Consistent with other qualitative studies on previous outbreaks, HPs faced numerous stressors, such as the fear of catching COVID-19 and spreading it to others; uncertainty about the virus, the disease, and the future; stigmatization, short-staffing, and inadequate PPE.^22,26,27^

Across separate cohorts of HPs in 2020, the level of moral injury increased dramatically, and functional impairment from moral injury symptoms nearly doubled.^14^ This qualitative study adds additional context to these numbers; the emotions reported—isolation, alienation, betrayal—significantly overlapped with the symptoms of moral injury in military and veteran populations.^28^ Some respondents who had expressed these emotions were so distressed to the point where their entire lives had changed dramatically, whereas others who have experienced the same events were disturbed but not as affected in their functioning.

In our previous study,^14^ we reported no change in burnout despite the increase in moral injury; this suggests that they may be parallel constructs. Indeed, in the qualitative analysis, experiencing moral injury was not associated with dehumanizing patients, one of the elements of burnout. Many spoke of how heartbroken they were that their patients were sick and/or dying alone, saying good-bye to their families virtually. It was in these moments that frontline workers demonstrated an enduring connection to their vocations in medicine.

### Limitations

Although we collected a large number of responses from HPs in various disciplines, several important limitations should be noted. The themes reported reflect the experiences of those who completed the survey and may not be generalizable. Of note, 487 survey respondents chose not to answer any of the qualitative questions, which may be attributed to survey burden. Our survey was only available in English, limiting responses to those who are fluent in it, and many of our respondents worked in North America. Nevertheless, our study’s results are trustworthy, as similar themes have been noted in qualitative studies in China^22^ and Italy.^26^ Although the COVID-19 pandemic represents an overarching morally injurious global event, we did not assess for regional, local, or individual morally injurious events that could have explained some of the variance in responses. Additionally, this was not a longitudinal study; as such, we could not definitively determine change over time or factors associated with change among individual respondents.

## Conclusions

This qualitative study discerned the themes of fear, fatigue, isolation, alienation, betrayal, and community among HPs in the prevaccine period of the COVID-19 pandemic. Respondents did not only point to specific morally injurious events; some described morally injurious environments in which they carried the burdens associated with being on the frontlines. This is consistent with other studies assessing the impact of organizational environment and culture on HP moral injury, distress, and burnout.^23,29^

The themes identified serve as potential starting points for organizations to use to engender and enhance organizational and individual recovery, team building, and trust. When leadership recognizes and openly acknowledges the prevalence and magnitude of moral distress, we can begin to move beyond slogans like “heroes work here” and the detrimental effects of military rhetoric.^30^ Systems that many survey respondents described as “broken” can be mended through initiatives to create environments that are in touch with new realities and what will likely be lasting memories of providing care in a pandemic. Examples of institutional responses that address HP concerns have been proposed by Shanafelt et al^19^: providing adequate PPE, rapid access to occupational health with prompt evaluation and testing, paid time off if quarantine is necessary, and support for physical needs, such as access to healthy meals, lodging for individuals on rapid-cycle shifts, and support for childcare needs.^19^ These actions will make simple and genuine expressions of gratitude feel substantial and meaningful.

## Notes

Supplement. **eAppendix 1.** Exemplar Quotations for Theme 1, Fear to Fatigue **eAppendix 2.** Exemplar Quotations for Theme 2, Isolation and Alienation **eAppendix 3.** Exemplar Quotations for Theme 3, Betrayal/Community
