# Supplementary material for: Morally Injurious Experiences and Emotions of Health Care Professionals During the COVID-19 Pandemic Before Vaccine Availability
Source: JAMA Netw Open. 2021 Nov 24;4(11):e2136150. doi: 10.1001/jamanetworkopen.2021.36150 (PMC8613593; doi:10.1001/jamanetworkopen.2021.36150)
Supplement: Supplement. — eAppendix 1. Exemplar Quotations for Theme 1, Fear to Fatigue eAppendix 2. Exemplar Quotations for Theme 2, Isolation and Alienation eAppendix 3. Exemplar Quotations for Theme 3, Betrayal/Community [file jamanetwopen-e2136150-s001.pdf]

Supplemental Online Content

Song YK, Mantri S, Lawson JM, Berger EJ, Koenig HG. Morally injurious experiences and emotions of health care professionals during the COVID-19 pandemic before vaccine availability. *JAMA Netw Open.* 2021;4(11):e2136150. doi:10.1001/jamanetworkopen.2021.36150

eAppendix 1. Exemplar Quotations for Theme 1, Fear to Fatigue

eAppendix 2. Exemplar Quotations for Theme 2, Isolation and Alienation

eAppendix 3. Exemplar Quotations for Theme 3, Betrayal/Community

This supplemental material has been provided by the authors to give readers additional information about their work.

© 2021 Song YK et al. *JAMA Network Open.*

eAppendix 1. Exemplar Quotations for Theme 1, Fear to Fatigue

1. I have COVID fatigue. I am borderline to the point of not caring. I used to be a lot more worried about it but I am not very much now. I can't be at anxiety level 10/10 for 7 straight months.

2. Everything was chaotic and scary in March, work policies were changing every week, leadership was inconsistent, PPE was a problem. Now we are adapting to the new normal because we are able to figure out what that is.

3. [A source of fear was] having to chart as an ICU nurse when I haven't practiced as one for 17 years. Getting in there and doing it fixed most of the fear. Covid-19 was not my biggest fear. I protect myself the best I can. I do not want to be the source of illness or death for my family or friends.

4. The greatest source of fear has been contracting COVID myself. I've become really kind of obsessive over cleaning everything I touch and washing my hands and not touching my face. I know I project that obsession on my husband. Everything I've learned about the virus says that there isn't much modern medicine can do to help. A ventilator isn't the answer. ECMO isn't going to be offered everywhere. I'm a healthy young person but that doesn't seem to matter given the cases I've read about where young people are just as ill. It's shaken my faith in medicine, it makes me feel vulnerable and scared to be honest. It also makes me scared to give it to my husband, my parents or anyone really. I have a lot of family and friends in New Jersey, and many of them have contracted it and been surprisingly ill. My great aunt passed away from it in a nursing home, alone, as they wouldn't let her daughters visit. She was a nurse in her career. The novelty of this virus and the range of acuity in symptoms among populations makes it really scary. Combined with no good way to treat it. I know what being intubated and sedated looks like, I know what dying alone looks like. I know that COVID patients are not getting the care we'd give, say, a person with flu, for the safety of the HCWs. I don't want to be that patient - isolated and somewhat neglected - and my care team slightly afraid of me. I don't want that for me, I don't want that for anyone.

5. For nurses PPE is constantly changing and what was necessary one day is not the next and vice versa, that makes it difficult to know whether we are adequately protected. We all just assume we will get sick or have asymptomatic COVID at some point. We also don't trust our POC testing and are fairly certain that patients with negative results do have it they just aren't coming back positive, increasing our risk. Also no one wants to take ownership of these COVID patients, MDs and RTs radio from the door, certain units refuse to take the patient and thus it becomes just the nurse put ourselves in harm's way while everyone else watches from afar and refuses to help in order to "lessen exposure." We are having to take on much more responsibility and far less support.

eAppendix 2. Exemplar Quotations for Theme 2, Isolation and Alienation

1. I am more distant with everyone. I avoid friends and family for fear of infecting them or exposing them to this as every day I feel that I am a carrier. I actively avoid my wife and now find ways to sabotage our relationship as my depression has hit new levels and I want her to leave and not witness this downward spiral.

2. My community is a small one, a small island just off the coast of San Diego, and we had this community Facebook page for updates and happenings and it has become this awful battleground of mask wearers vs non mask wearers and people who want to open the beach vs not.... the whole thing is a big political mess all of the sudden which makes me even sadder.

3. My biggest problem at this point is dealing with deniers and anti-maskers and the politicizing of what should just be a humanitarian concern. It effects my life directly and hurts more than I'd like to admit and is difficult to separate so I deal with anger I am not used to carrying. Social media has been stressful, it is most of my social interaction outside of work since I like with a high-risk individual, but the bombardment of deniers fueled by [President Trump] and others is so frustrating after I have dealt with death or even just the difficulty of caring for these patients. Even seeing their families denying that it could be the cause it is mind boggling and shouldn't be the case.

4. We are all wearing masks. Sometimes I don’t even recognize my coworkers in the hallway. I am a fairly affectionate person - but now conscious of not touching anyone and feeling a bit guilty when I reach out without thinking. Also with patients, it makes me crazy not to take a hand when patient extends for prayer. Not to embrace those patients and family members for which this is the norm or expected. Standing farther away from a colleague than previously - it is stressful. Around the office feeling tingle of guilt when I have removed my mask in my space and someone comes in, or if I walk out into the shared area and forget to mask.

5. I miss close interaction with coworkers without masking (like lunch time & chats or potlucks which always seemed to lift spirits). I'm not able to play Volleyball right now, which was always my therapeutic outlet for stress. Am really struggling to find the healthy balance of mental health & avoid depression. Feel like my life & everyone I encounter every day, is consumed by COVID whether it be discussing, analyzing or treating or rearranging (staff & scheduled appointments). I literally don’t know where I am working the next day until sometimes 8pm the night before or even the hours. It’s exhausting.

6. I’ve completely isolated myself due to the risk of exposing friends, family, and the public. I live alone now, and I moved closer to work so I don’t have to take public transportation & can walk. We are perpetually understaffed. I work 15h shifts, and sleep. My family is 3,000 miles away on the west coast. We try to do zoom meetings when we

can. Few people I know work in death investigation, and those who do don't want to talk about it much during their time off. Lost my 3 year relationship during pandemic. Work hours and time to decompress/ rest after shifts makes even a virtual social life almost impossible.

7. I personally held 11 hands in less than three months, as my patients slipped away. They died without family close, but I didn't let them die alone, I couldn't. No matter what else was happening, no one dies alone. All of my patients that passed away had been put on comfort care, thankfully, they avoided horrific and painful deaths. I was often the one to tell the family when my patient passed away. That had never been my job before. I graduated from nursing school at the end of February 2020. I've been a nurse for less than a year and have experienced more death than many seasoned nurses. I am an angel of death and comfort. That weighs on me.

8. My first COVID+ patient death stands out as my most painful experience during this outbreak. I generally enjoy providing end of life care to my patients and offering comfort to their families and friends. But in light of the no visitor policy, I found myself more involved in this death than any other before - holding my patient's hand in place of his wife of almost 50 years as she bawled on FaceTime. In the midst of the experience, I found myself taking on the blame for the policy that she was not allowed to be present for his death and powerless that I had no pull to get her there. As she cried, I cried quietly and out of sight too; it was hard not to take on her pain. But I also found that because I did not leave his room for hours, I could be fully focused on his dying process and available to meet every need to keep him comfortable and help maintain his dignity. I was not emotionally prepared for this experience, and it hurt so much more than any other patient death in my 10 years of critical care nursing.

eAppendix 3. Exemplar Quotations for Theme 3, Betrayal/Community

1. There was not proper PPE at the time. We were doing aerosolizing procedures without N95s, and management made me remove hospital issued safety goggles while screening patients because it was not being "good stewards of PPE" to use them for that task, but I was welcomed to bring my own in. They did not take the goggles back, just required they not be on my face. I actually sat my 17-year-old down and explained the life insurance, wills, last wishes, etc. after that week. I felt like our lives were more disposable than our PPE was. Since the summer, things have been much better. Our patients are screened before their procedures, we are required to wear N95s for our procedures, and I no longer isolate myself from my family at home.

2. I felt as though we were being “offered up for slaughter” by having to stay in a COVID filled room with questionable PPE. Then I felt guilty for thinking of myself. But then what if I got COVID; I have a family. And then I felt horrible for the patient, alone except for me - their nurse who was worried about herself. The back and forth guilt and worry about wanting to protect myself (and felt like a pawn in a game by hospital admin) and wanting to fully care for the pt was exhausting. I’m sure I’m not the only one who was feeling that way. Our chaplain was a wonderful resource, but we needed debriefing from our leadership. Instead of debriefing, they opted to spend money on “healthcare heroes” signs outside the hospital. It felt like a slap in the face. I became resentful towards admin/hospital leadership. It was as though it was taboo or uncomfortable for leadership to come and talk to us. It’s like when your friend is grieving and you don’t know what to say so you send meals and a card, but what your friend really needs is you there sitting next to them as they talk through what they’re mentally processing.

3. To the staff nurses that are on the floor, it strengthens our bond. I am having a hard time trusting management and the institution. While I know that some of the lack of PPE and safety measures are due to an actual shortage, I feel like institutions should be doing better for their front line staff. Upper management doesn't know what it's like to do this every time we come into work. It is starting to wear down a lot of people.

4. At first, we came together to really help each other out because we are trapped in these negative airflow rooms with no good way to call for help when we need it. Now, as the units have opened up to accommodate non-COVID, staff manipulate the staffing assignments so they don’t end up with COVID patients, which results in a handful of staff constantly assigned to COVID patients, making us feel even more isolated. Staff are leaving in droves, leaving us short staffed and flexed up with our patients, causing even more stress over the already heavy workload with COVID patients. We start to feel angst towards other disciplines because the bedside nurse has had to adopt many of the ancillary departments’ roles with COVID patients. Nurses and respiratory therapists remain the only two disciplines to enter COVID rooms, meaning we are doctor, nurse, dietary, housekeeping, phlebotomy and PT/OT. And we continue to have more roles dumped on us, so there is definitely resentment towards those that won’t enter the rooms.

5. We pulled staff from all over the hospital and dumped them in these makeshift units. Nurses that were from all different specialties were all put together and expected to team nurse. The stress was unreal and the unit was like a battlefield. I saw more morgue carts leave our unit than actual patients. It wasn't uncommon to lose more than one patient a shift.

   One day in particular sticks out to me. I was one of "the regulars" on this makeshift ICU in the Interventional Radiology area. I was put alongside non-ICU nurses, acting as charge nurse, and also had 3 patients of my own. The day started with holding a patient's hand as he passed away without family by his side. Progressed to proning a crashing patient, intubated a patient that could no longer hold out on max oxygen settings, and another patient's blood sugar was 20. With all of this, I was also caring for a patient whom I had on and off for weeks. I got to know him pretty well. When I go to work this particular morning, I knew something was wrong. I made sure to spend extra time talking to him, sat down next to him in my full PPE so it didn't look like I was rushed. As he declined, I spoke with him about options. I promised I would hold his hand. We cried together.

   The docs wanted me to help him prone himself. I knew it wasn't going to end well so I asked the docs to stand by with the cart. As I went in and they all stood at the glass door, I felt like I was it – alone – and everything. I explained to the patient what the plan was and what would happen if it didn't work. The panic in his face and eyes still haunt me. When proned, he quickly deteriorated. I looked at the docs through my PPE and gave them the nod to call the protected respiratory code. I explained to him how people would come in, what would happen, how I would be by his side holding his hands. At this point in COVID, intubation was no longer a bridge to save people; it was a one-way ticket to death. I knew he wouldn't survive. As anesthesia came in, I grabbed the patient's phone and told him to call his son. I looked at the anesthesiologist expecting to get yelled at, but he gave a nod and told the patient it was ok. Everyone in that room knew what I was doing – this was going to be the last time he would ever talk to his son. And it was. After intubation he quickly deteriorated and died 2 days later. I left work at 21:45 that night. Never had a break, never had a meal or snack. I cried so hard the whole drive home. I called off the next day. I couldn't get out of bed. It's a day that will forever haunt me. And I have many other days that do too.

© 2021 Song YK et al. *JAMA Network Open.*
